# Supplementary material for: Revealing the order parameter dynamics of 1T-TiSe2 following optical excitation
Source: Sci Rep. 2022 Sep 23;12:15860. doi: 10.1038/s41598-022-19319-w (PMC9508156; doi:10.1038/s41598-022-19319-w)
Supplement: Supplementary file 1 — Supplementary Information. [file 41598_2022_19319_MOESM1_ESM.pdf]

Supplementary Information

Maximilian Huber [1], Yi Lin [1], Nicholas Dale[1,2], Renee Sailus[3], Sefaattin Tongay[3], Robert A. Kaindl [1,4] and Alessandra Lanzara[1,2*]

[1]Materials Science Division, Lawrence Berkeley National Laboratory, Berkeley, CA 94720, USA
[2]Physics Department, University of California Berkeley, Berkeley, CA 94720, USA
[3] Materials Science and Engineering Department, Arizona State University AZ 85281, USA
[4] Department of Physics and CXFEL Labs, Arizona State University, AZ 85287, USA

*To whom correspondence should be addressed; E-mail: alanzara@lbl.gov

Methods

Time-resolved ARPES measurements were conducted at the Lawrence-Berkeley National Laboratory with 22.3 eV extreme-ultraviolet (XUV) femtosecond pulses. Photoelectrons are detected with a hemispherical electron analyzer (Scienta R4000). The light source was a cryo-cooled regenerative amplifier (KMLabs Wyvern 500) seeded by the output of a home-built, 76 MHz Ti:sapphire oscillator, which is pumped by 4.5 W from a green solid-state laser (Lighthouse Photonics Sprout). The amplifier stage, in turn, is pumped by two green, nanosecond pulsed Nd:YVO_4 lasers (Photonics Industries DS20HE). The XUV is created after second harmonic generation by tightly focussing 390 nm pulses into Kr gas. A detailed description of the setup can be found in (*1, 2*). For the high fluence experiment (80 $\mu J/cm^2$) a pump wavelenght of 780 nm with a repetition rate of 50 kHz was used with a total energy resolution of ∼80 meV and a temporal resolution of about 65 fs. For all the other data sets a repetition rate of 25 kHz was used.

Supplementary Note 1: Raw data processing

To find time zero, excited carrier dynamics (integration window is shown in Figure S1) were fitted and the parameter t_0 extracted. This provides a consistent determination across the set of

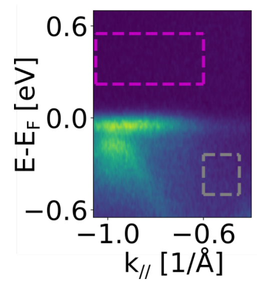

Figure S1: ARPES spectrum at -1 ps delay. Boxes show the integration regions used for normalization (grey) and determination of time zero (magenta).

various measurements.
To account for fluctuations in laser intensity during the experiment, ARPES spectra were normalized to an area between the high symmetry points with no band structure. The region where the background is taken is shown in Figure S1 marked by the grey box. For the relative changes in the Se_{4p}* band intensity this background was subtracted from the intensity of the Se_{4p}* band.

Supplementary Note 2: Doping Dependence

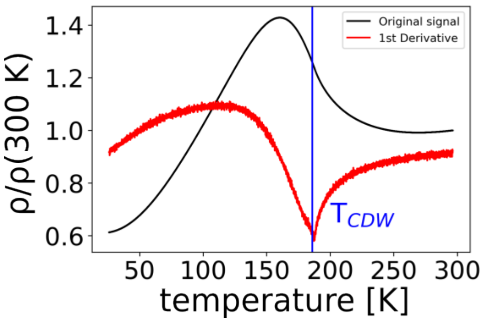

Figure S2: Temperature dependent resistivity (black) as well as first derivative (red) of the sample measured with 80 $\mu J/cm^2$.

For this work two different batches of samples were used which varied slightly in the amount of Ti self-doping. The sample measured with 80 $\mu J/cm^2$ came from the first batch, whereas the samples measured with all other fluences came from the second batch. The first batch of $TiSe_2$ samples had a slightly higher self-doping, which explains the different Ti_{3d}/Se_{4p}* intensity ratio seen in the EDCs in Figure 2 of the main text (*3*). Due to this difference in doping, the transition temperature of the first batch of samples are slightly lower (∼187 K instead of ∼ 203 K for the batch of samples with a very low doping level), as evidenced by the resistivity curve shown in Figure S2. Nevertheless, this leads only to a negligible difference in the

equilibrium gap size which is within the error bars of our experiment (*3*).

Supplementary Note 3: Gap fitting

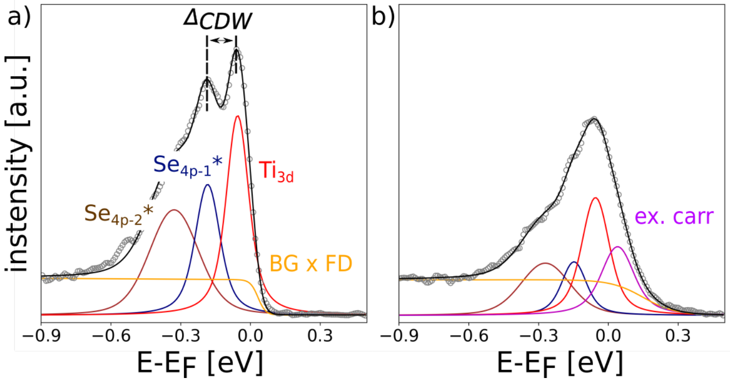

Figure S3: a) Exemplary fitted EDC at M point at a delay of -1 ps. Circles represent data points, the solid black line is the best fit result. Also shown are the fitted peaks, corresponding to Ti_{3d} (red), Se_{4p-1}* (blue) and Se_{4p-2}* (brown) band, respectively. The orange line represents the linear background multiplied with the Gaussian broadened Fermi-Dirac distribution. b) Fitted spectrum at delay 210 fs. The additional magenta peak accounts for an additional band populated by the excited carriers.

To extract the gap size, EDCs taken at M point were fitted using three peaks of Voigt line-shape, accounting for the Ti_{3d} band as well as the two spin-orbit split Se_{4p-1}* and Se_{4p-2}* bands, on a linear background. All of these components are multiplied with a Gaussian-broadened Fermi-Dirac distribution function. An exemplary fit is shown in Figure S3a. To account for the transient population of in equilibrium unoccupied states above the Fermi level and the increase in electronic temperature, the width of the Fermi-Dirac distribution as well as the center of the Fermi level was allowed to vary. We note that all EDCs consistently show a shoulder at around -0.5 eV, which could originate from a folded Se_{4p-z} (*4*) orbital. Indeed, the spectra can be slightly better fitted when adding another peak, however for simplicity we decided to fit with three peaks. The gap dynamics are highly identical for all fluences on all samples whether a fourth Se_{4p-z} peak is used or not. For the high fluence data (78 and 80 $^{\mu J}/_{cm^2}$) the system gets considerably driven out of equilibrium and a large amount of hot electrons gets excited, so that it becomes necessary to include a fourth peak at intermediate delay values above the Fermi level (see magenta peak in panel b). This peak can be assigned to a higher lying Ti_{3d} conduction band (*5, 6*), which is discussed in more detail in (*7*). For the Voigt peaks and the broadened Fermi level the Gaussian contribution was set to be the experimental energy resolution (80 meV). For the data set where the Γ point was simultaneously recorded (80 $^{\mu J}/_{cm^2}$), the peak positions for the Se_{4p-2}* band were fixed to be within a range of $\pm$20 meV of the positions

obtained from the fits at Γ point, since the binding energy of the folded Se_{4p-2}* is identical to the one of the main Se_{4p-2} band.

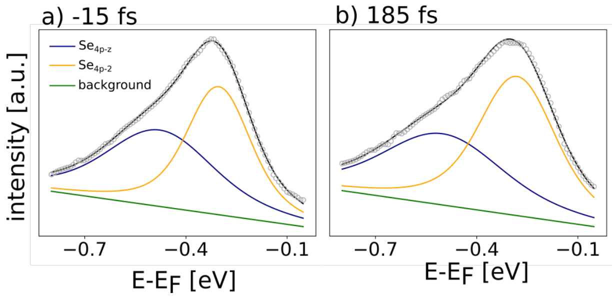

Figure S4: Fitted EDCs taken at Γ point at -1 ps and 185 fs, respectively.

As the Se_{4p} band barely decreases in intensity at the Γ point, the valence band position can be extracted with high accuracy. EDCs taken at Γ point were fitted using two peaks (accounting for the Se_{4p-2} and a Se_{4p-z} (*4*) band) of Voigt lineshape on a linear background. A third peak, accounting for the Se_{4p-1} orbital can be included, but does not significantly improve or influence the fit so that it was left out.

For the gap fits the FWHM and amplitude of the two spin split Se_{4p-1}* and Se_{4p-2}* bands were set to have constant ratios ($\pm 20\%$) over all delays. The position of the lowest Ti_{3d} band is in close vicinity to the Fermi level and thus a source of potential errors in the fits, especially for spectra taken after stronger excitation when the systems gets driven strongly out of equilibrium. Therefore, we fixed its center to be within a range $\pm 25 meV$ of the equilibrium position for all delay values, which is a reasonable choice as it does not hybridise with the Se_{4p}* band (*5*) and thus should show a similar binding energy in the high and low symmetry phase. Temperature dependent studies found an upshift of ~25-35 meV (*8, 9*) when going from 100 K to 300 K, however caused by an shift of the chemical potential. In a leading edge analysis of our data we do not observe an upshift of the peak corresponding to the Ti_{3d} band even after strong excitation. Thus we conclude that fixing the Ti_{3d} peak position within a close range only leads to negligible error in the obtained fitting results for the gap size. Furthermore if there was an actual pronounced upshift of the Titanium band it would mean that the actual gap size is even larger (and the quench of the gap size even smaller) than obtained from our fits, in line with our main result of a robust gap. The absence of an upshift of the Ti_{3d} band also allows us to exclude significant influence of pump induced space charge effects on the data.

Due to the high number of parameters in the fitting function, the values of the initial guesses for the peak positions were systematically varied to achieve the best fit results. For all fluences additional gap fits were performed at slightly different positions in momentum space to reassure the obtained results. To furthermore check the quality of the fits, the ratio of the peak amplitudes

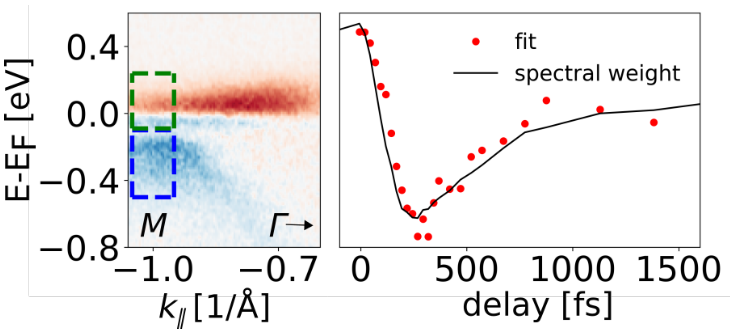

Figure S5: a) Difference spectrum between 270 fs and -1 ps after excitation with 80 $\mu J/cm^2$. Red marks an increase, blue and decrease of intensity. b) Ratio of the integrated blue and green area from panel a (black curve) compared to the peak ratio of the corresponding peaks in the EDC fits (red circles).

corresponding to the Ti_{3d} and Se_{4p-1}* and Se_{4p-2}* (and for the high fluence data the peak accounting for the excited carrier population) multiplied by the fitted Fermi-Dirac distribution was compared to the ratio of the integrated intensity at energy locations corresponding to these peaks. As one can see in Figure S5, a good correlation can be found. Error bars for the data taken with 80 $\mu J/cm^2$ were calculated using the Markov Chain Monte Carlo method, for all other data sets they were calculated by bootstrapping. The absolute values of the errorbars are given for a 95% confidence interval in both cases.

Supplementary Note 4: Additional gap data

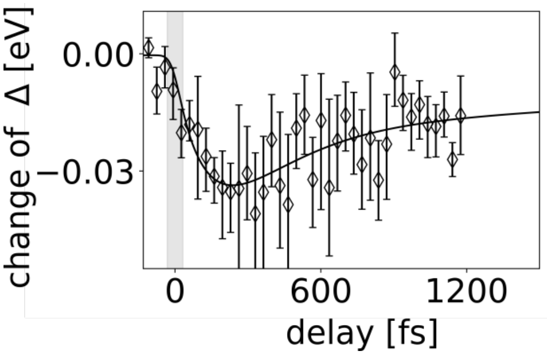

Figure S6: Gap Dynamics after excitation with 78 $\mu J/cm^2$.

For completeness in Figure S6 we also show the gap dynamics after excitation with 78 $\mu J/cm^2$ extracted along Γ-M-Γ direction.

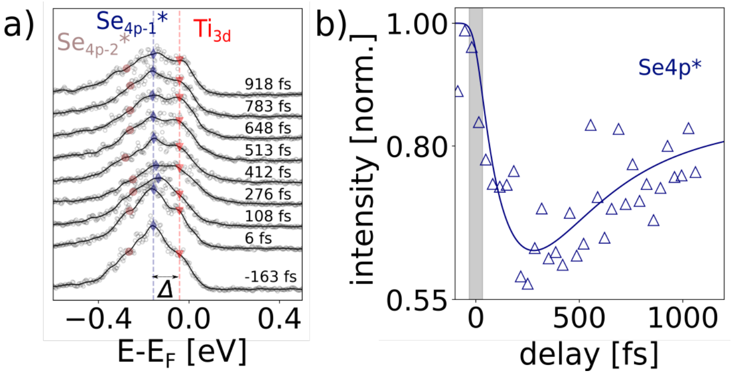

Figure S7: a) Selected EDCs taken at M point with markers indicating the fitted peak positions of the Ti_{3d}-a conduction band (red triangle) and the Se_{4p-1} (blue diamonds) and Se_{4p-2} valence bands (brown circles) after excitation with 90 $^{\mu J}/_{cm^2}$. Circles represents raw data and solid black lines represent the smoothed raw data using the Gaussian method (20 meV window). b) Integrated intensity of the folded Se_{4p}* band at M point for spectra taken along $K - M - K$ direction after excitation with 90 $^{\mu J}/_{cm^2}$.

To confirm the results of the gap fits in Figure 2 of the main paper, we also extracted the gap dynamics at M for spectra taken along the less studied K-M-K direction. The resulting EDCs after excitation with 90 $^{\mu J}/_{cm^2}$are shown in Figure S7 a. They clearly show, despite the strong excitation with 90 $^{\mu J}/_{cm^2}$, only a marginal decrease of the gap size, with the gap being clearly open for all delay times. Note that due to matrix element effects the intensity of the Se_{4p}* valence bands is sensitive to different polarizations of the XUV probe. To optimize the photoemission signal from the Se_{4p}* bands, we used a s-polarized probe for spectra along Γ-M-Γ direction and a p-polarized probe for spectra along $K - M - K$ direction. Despite the different probe polarizations the EDCs taken along both high symmetry direction are almost identical and the equilibrium gap size is the same within the experimental error bars. To further confirm that data taken along K-M-K is comparable with the spectra taken along Γ-M-Γ direction, we also show the dynamics of the Se_{4p}* folded band along K-M-K. Indeed, the curve shows a almost identical behavior, in terms of dynamics as well as extent of quench, to the data taken after excitation with 80 $^{\mu J}/_{cm^2}$ along Γ-M-Γ shown in Figure 3 of the main paper. These results give us further confidence in our assessment that the gap does only marginally close within the fluence range studied in this work.

Supplementary Note 5: Fitting of delay curves

All curves which display a the dynamic over time can be fitted using a function of the form:

$$I(t) = \Theta \cdot (-a_{build} \cdot exp(-(t-t_0)/\tau_{build}) + a_{fast} \cdot exp(-(t-t_0)/\tau_{fast})$$
$$+ a_{slow} \cdot exp(-(t-t_0)/\tau_{slow})) + C$$

with Θ being the Heavyside step function, $a_{build,fast,slow}$ the amplitudes, τ_{build} the time constants for build-up time and τ_{fast} and τ_{slow} the timescales of the fast and slow recovery component, respectively. C is a constant offset. The whole function $I(t)$ is then convoluted with a Gaussian distribution function to account for the temporal resolution of the experimental setup (65 fs (*1*)). Note that in order to to ensure comparability between the different fits we constrained τ_{slow} to be within a range of 4-5 ps for all data sets.

Supplementary Note 6: Correlations at different fluences

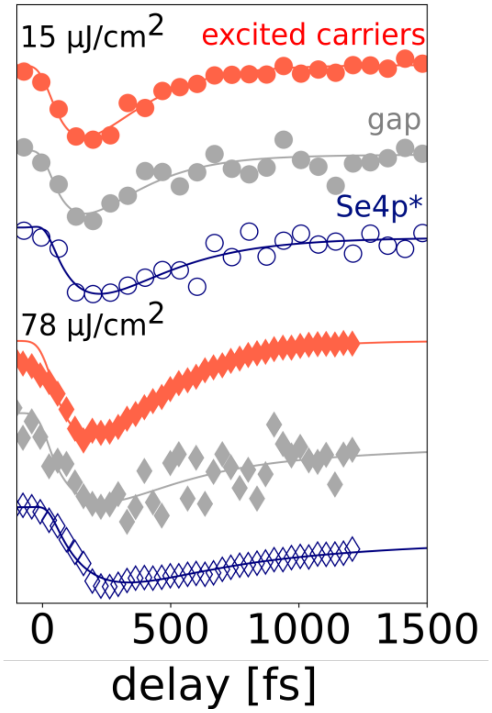

Figure S8: Correlation between excited carriers, gap and Se_{4p}* spectral weight for 15 and 78 $^{\mu J}/_{cm^2}$.

Supplementary Note 7: Pathway of excited carriers

Figure S8 shows the dynamics of the excited carriers at 0.6 eV and above the Fermi level extracted from the integrated intensity in the green and red boxes shown in the inset, respectively. Right after pump excitation the carriers are excited all the way up to 0.6 eV (see green curve), in correspondence to the unoccupied Ti_{3d} band, and reach their maximum within a few femtoseconds, to then quickly decay towards equilibrium in less than 500 fs. This result is in agreement with previous reports where the excitation from the $Se4p_{x,y}$ orbital into a Ti_{3d} band at this energy level has been assigned to the main photoexcitation pathway (*10*). In contrast, the excited carriers right above the Fermi level (red curve) reach their maximum much later after about 200 fs when the higher energy excited carrier population is almost back to equilibrium, suggesting a scattering of excited carriers from this high energy toward the unoccupied states right above E_F. Thus, the build up time of the red box gives a measure of the scattering time of originally to higher energy level excited electrons down to the Fermi level. As only the excited carrier population close to the Fermi level shows a good correlation with the gap and in thus of major

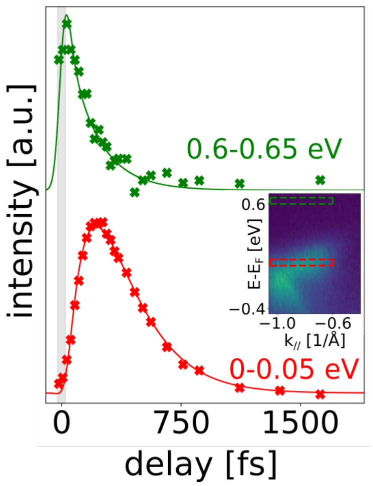

Figure S8: Integrated intensity 0.6 eV (green data points) and right above (red data points) the Fermi level after excitation with 80 $\mu J/cm^2$. Integration regions are shown in the inset. Solid lines represent fits to the data points.

interest for this work, other integration regions are neglected in the main paper. A momentum dependence of the hot electron dynamics cannot be seen.

References

1. J. H. Buss, H. Wang, Y. Xu, C. Jozwiak, J. Pepper, J. Maklar, F. Joucken, L. Zeng, S. Stoll, Z. Hussain, A. Lanzara, Y.-d. Chuang, J. D. Denlinger, H. Wang, Y. Xu, J. Maklar, L. Zeng, J. Pepper, and Y.-d. Chuang, "A setup for extreme-ultraviolet ultrafast angle-resolved photoelectron spectroscopy at 50-kHz repetition rate," *Review of Scientific Instruments*, vol. 90, no. 023105, 2019.

2. H. Wang, Y. Xu, S. Ulonska, J. S. Robinson, P. Ranitovic, and R. A. Kaindl, "Bright high-repetition-rate source of narrowband extreme-ultraviolet harmonics beyond 22eV," *Nature Communications*, vol. 6, no. 7459, 2015.

3. T. Jaouen, B. Hildebrand, M. L. Mottas, M. Di Giovannantonio, P. Ruffieux, M. Rumo, C. W. Nicholson, E. Razzoli, C. Barreteau, A. Ubaldini, E. Giannini, F. Vanini, H. Beck, C. Monney, and P. Aebi, "Phase separation in the vicinity of Fermi surface hot spots," *Physical Review B*, vol. 100, no. 7, pp. 1–11, 2019.

4. M. Cazzaniga, H. Cercellier, M. Holzmann, C. Monney, P. Aebi, G. Onida, and V. Olevano, "Ab initio many-body effects in $TiSe_2$: A possible excitonic insulator scenario from GW band-shape renormalization," *Physical Review B - Condensed Matter and Materials Physics*, vol. 85, no. 19, pp. 1–6, 2012.

5. M. D. Watson, O. J. Clark, F. Mazzola, I. Marković, V. Sunko, T. K. Kim, K. Rossnagel, and P. D. King, "Orbital- and kz -Selective Hybridization of Se 4p and Ti 3d States in the Charge Density Wave Phase of $TiSe_2$," *Physical Review Letters*, vol. 122, no. 7, pp. 1–6, 2019.

6. C. Monney, H. Cercellier, F. Clerc, C. Battaglia, E. F. Schwier, C. Didiot, M. G. Garnier, H. Beck, P. Aebi, H. Berger, L. Forró, and L. Patthey, "Spontaneous exciton condensation in $1T-TiSe_2$: BCS-like approach," *Physical Review B - Condensed Matter and Materials Physics*, vol. 79, no. 4, pp. 1–11, 2009.

7. M. Huber, Y. Lin, N. Dale, R. Sailus, S. Tongay, R. A. Kaindl, and A. Lanzara, "Mapping the dispersion of the occupied and unoccupied band structure in photoexcited 1T-$TiSe_2$," *Journal of Physics and Chemistry of Solids*, vol. 168, no. 110740, 2022.

8. K. Rossnagel, L. Kipp, and M. Skibowski, "Charge-density-wave phase transition in (formula presented): Excitonic insulator versus band-type Jahn-Teller mechanism," *Physical Review B - Condensed Matter and Materials Physics*, vol. 65, no. 23, pp. 1–7, 2002.

9. C. Monney, E. F. Schwier, M. G. Garnier, N. Mariotti, C. Didiot, H. Beck, P. Aebi, H. Cercellier, J. Marcus, C. Battaglia, H. Berger, and A. N. Titov, "Temperature-dependent photoemission on 1T -$TiSe_2$: Interpretation within the exciton condensate phase model," *Physical Review B - Condensed Matter and Materials Physics*, vol. 81, no. 15, pp. 1–9, 2010.

10. G. Rohde, T. Rohwer, A. Stange, C. Sohrt, K. Hanff, L. X. Yang, L. Kipp, K. Rossnagel, and M. Bauer, "Does the excitation wavelength affect the ultrafast quenching dynamics of the charge-density wave in $1T-TiSe_2$?" *Journal of Electron Spectroscopy and Related Phenomena*, vol. 195, pp. 244–248, 2014.
